# Computational measurement of tumor immune microenvironment in gastric adenocarcinomas

**DOI:** 10.1038/s41598-018-32299-0

**Published:** 2018-09-17

**Authors:** Young Hwan Chang, You Jeong Heo, Junhun Cho, Sang Yong Song, Jeeyun Lee, Kyoung-Mee Kim

**Affiliations:** 1 Department of Biomedical Engineering and Computational Biology Program, Oregon Health and Science University (OHSU), Portland, OR 97239 USA; 2 The Samsung Advanced Institute for Health Sciences & Technology (SAIHST), Samsung Medical Center, Sungkyunkwan University School of Medicine, Seoul, Korea; 3 Department of Pathology & Translational Genomics, Samsung Medical Center, Sungkyunkwan University School of Medicine, Seoul, Korea; 4 Division of Hematology-Oncology, Department of Medicine, Samsung Medical Center, Sungkyunkwan University School of Medicine, Seoul, Korea

## Abstract

The use of four groups of tumor immune microenvironments (TME) based on PD-L1 and tumor-infiltrating T lymphocytes (TIL) is a reliable biomarker for anti-PD-1/PD-L1 inhibitor therapy. We classified the TME in 241 gastric cancers which were subdivided according to 40 EBV+, 76 microsatellite instability-high (MSI-H), and 125 EBV-/microsatellite-stable (MSS) subtypes by quantitative image analysis (QIA) and correlated the results with mRNA expression levels. The mean PD-L1 ratio and CD8 ratio in EBV+, MSI-H, and EBV−/MSS GCs were significantly different (P < 0.006). The PD-L1 ratio and CD8 ratio obtained by QIA correlated well with the RNA levels of PD-L1 (r = 0.63) and CD8 (r = 0.67), respectively. The TME were type I (PD-L1^H^/CD8^H^) in 45, type II (PD-L1^L^/CD8^L^) in 106, type III (PD-L1^H^/CD8^L^) in 8, and type IV (PD-L1^L^/CD8^H^) in 82 cases. The type I TME was significantly associated with high TIL (P = 3.0E-11) and EBV+ status (P = 8.55E-08). In conclusion, QIA results correlated well with the mRNA expression levels and classified TME of gastric cancers.

## Introduction

Precision cancer treatment depends not only on next-generation sequencing (NGS), but also on visual assessment of protein biomarker expression seen on immunohistochemistry (IHC) slides. Pathologists interpret IHC results, a difficult and time-consuming task. However, the final IHC results are subjective and qualitative in nature. There is inter-observer variability, and this method is not well suited to the evolving landscape of biomedical research^1^. This variability among pathologists, coupled with the inherent heterogeneity seen among cancers, suggests that a more objective and truly quantitative strategy is the true gold standard toward which the biomedical community should strive^2^. Therefore, the advent of high-throughput acquisition technologies, such as automated slide scanners and computerized analysis of tissue images, is highly desirable. Studies have shown that quantitative software can detect changes in disease states that are missed by visual inspection^3^.

The tumor immune microenvironment (TME) is increasingly recognized as a key factor in multiple stages of disease progression, particularly local resistance, immune-escaping, and distant metastasis. The TME substantially impacts the future development of frontline interventions in clinical oncology^4^. The use of four TME groups based on PD-L1 and tumor-infiltrating lymphocytes (TIL) status is a reliable biomarker for anti-PD-1/PD-L1 inhibitor therapy. Immunohistochemistry (IHC) is currently used to detect PD-L1 on tumor cells and immune cells. However, positivity and clinical significance vary due to the type of specimen used (whole histological sections vs. tissue microarrays), type of PD-L1 primary antibodies, and pathological interpretation method^5–7^. The addition of immune cellular markers to PD-L1 IHC would provide deeper insight into the understanding of the complex TME^8^.

Recently, immunotherapy has become a promising approach to treat GC^9^. We hypothesized that if a full-section quantitative image analysis (QIA) method can classify TME subtypes, it can be used as a predictive biomarker for anti-PD-1/PD-L1 inhibitor therapy with high reproducibility. For this purpose, we performed IHC for PD-L1 and CD8+ T cells in three distinct subtypes of gastric cancer (GC) (EBV+, MSI-H, and EBV−/MSS), interpreted them with QIAl and correlated the results with mRNA expression levels.

## Results

### Quantitative image analyses (QIA) of PD-L1 and CD8 IHC

**Figure 1:**
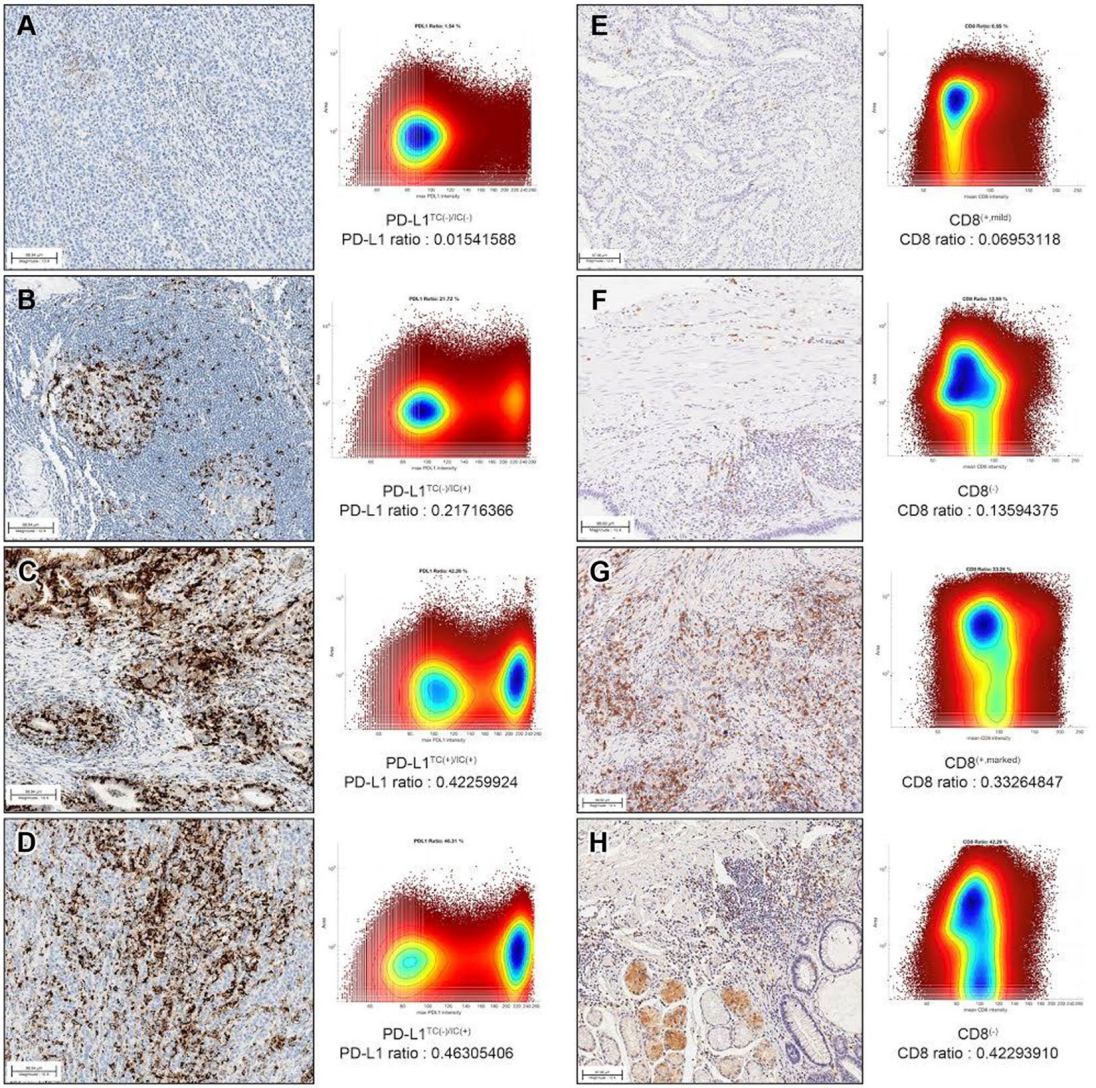
Representative images of PD-L1 (**A**–**D**) and CD8 (**E**–**H**) immunohistochemistry. PD-L1 ratio (**A**–**D**) and CD8 ratio (**E**–**H**) were obtained from computational analyses.

In QIA of whole slide imaging, representative images of PD-L1 (Fig. 1A–D) and CD8 (Fig. 1E–H) IHC, with paired bivariate scatter plots for both cell size (area) and intensity values, are provided in Fig. 1, where each dot represents a single cell. In the representative whole IHC-stained slides from each GC case, the mean number of PD-L1-positive cells was 206,132 (range: 12,415-696,449), and the mean number of total cells on a slide was 1,555,897 (range: 26,065–2,821,182). The mean PD-L1-ratio (absolute number of PD-L1-positive cells per total number of cells) was 0.1387 (range: 0.0154–0.4763). The mean number of CD8-positive cells was 532,128 (range: 142,374-1,716,791), and the mean number of total cells within the slide was 2,197,705 (range: 622,033–4,388,177). The mean CD8 ratio was 0.2332 (range: 0.0695–0.4876).

The QIA results regarding PD-L1 ratio, PD-L1 count, CD8 ratio, and CD8 count in 40 EBV+, 76 MSI-H, and 125 EBV(−)/MSS GCs are described in Table 1. The mean (standard deviation: SD) PD-L1 ratios in EBV+, MSI-H, and EBV−/MSS GCs were 0.19 (0.13), 0.10 (0.08), and 0.06 (0.06), respectively, and this difference was significantly different (P < 0.001). The mean (SD) CD8 ratios in EBV+, MSI-H, and EBV−/MSS GC were 0.25 (0.14), 0.21 (0.12), and 0.18 (0.11), and these differences were significant (P = 0.006).

**Table 1:** Results of quantitative image analysis in three distinct subtypes of gastric carcinomas.

	EBV+	MSI-H	EBV−/MSS	p value
**QIA mean values (standard deviation)**				
PD-L1 ratio	0.19 (0.13)	0.10 (0.08)	0.06 (0.06)	<0.001
PD-L1 counts	308789.22 (262974.48)	142755.72 (124169.97)	92239.30 (100996.94)	<0.001
CD8 ratio	0.25 (0.14)	0.21 (0.12)	0.18 (0.11)	0.006
CD8 counts	382905.08 (272642.21)	268919.76 (192954.09)	240017.42 (208248.02)	0.002
Number of cases	40	76	125	

Interestingly, although the PD-L1 ratio and count were significantly higher in both the EBV+ and MSI-H GC groups, the CD8 ratio and CD8 count were significantly higher in the EBV+ GC group, but not in the MSI-H GC group, compared to the MSS GC group. This result suggests that CD8+ cells heavily infiltrate both EBV+ and MSI-H GC, but there is a lower degree of infiltration in MSI-H GC compared to EBV+ GC.

### Comparison of QIA to mRNA expression and clinicopathological variables

**Figure 2:**
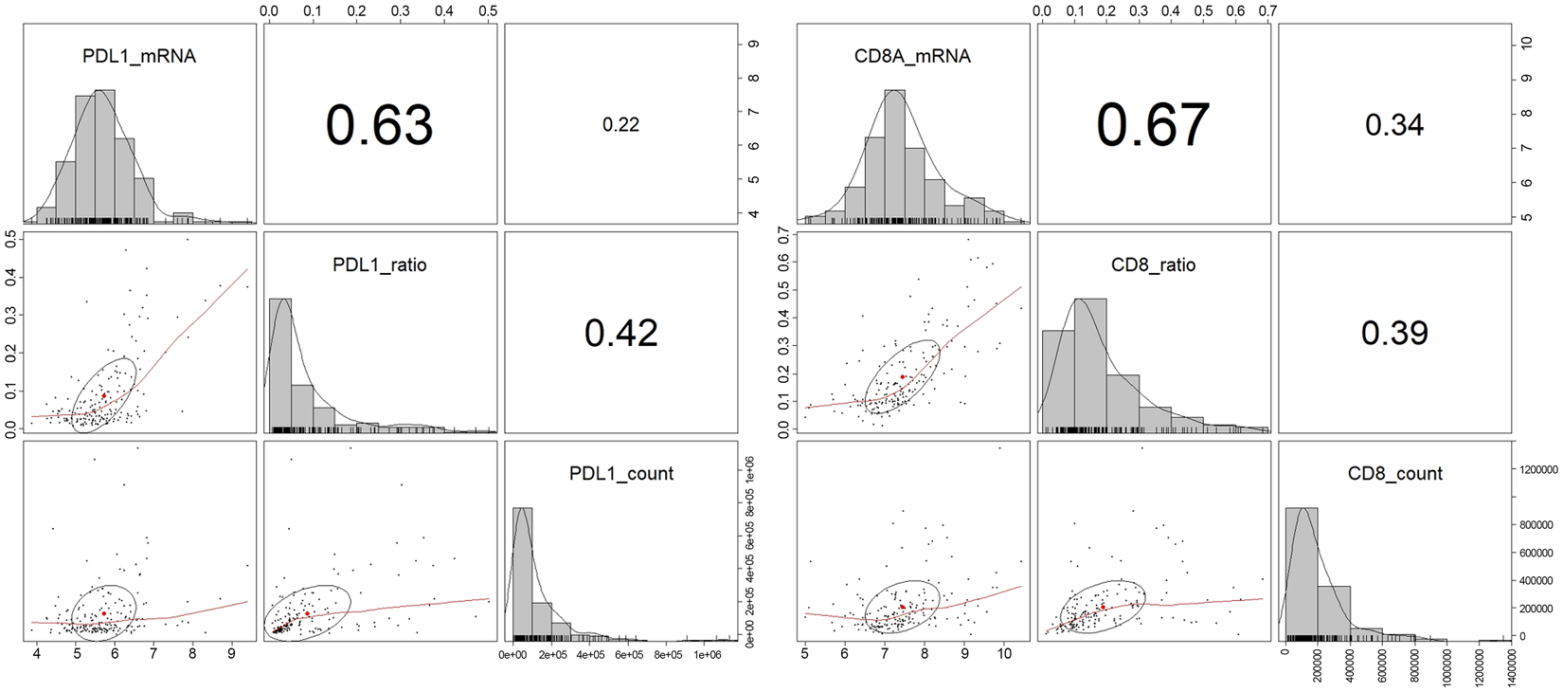
Direct comparison of mRNA expression level and quantitative image analysis results. PD-L1 ratio showed higher correlation with PD-L1 mRNA than PD-L1 count, and CD8 ratio had higher correlation with CD8 mRNA than CD8 count.

Direct comparison of mRNA expression levels and QIA results showed a higher correlation between PD-L1 mRNA expression level and PD-L1 ratio (r = 0.63) than with PD-L1 count (r = 0.22). CD8 mRNA was more highly correlated with CD8 ratio (r = 0.67) than CD8 count (r = 0.34) (Fig. 2).

**Figure 3:**
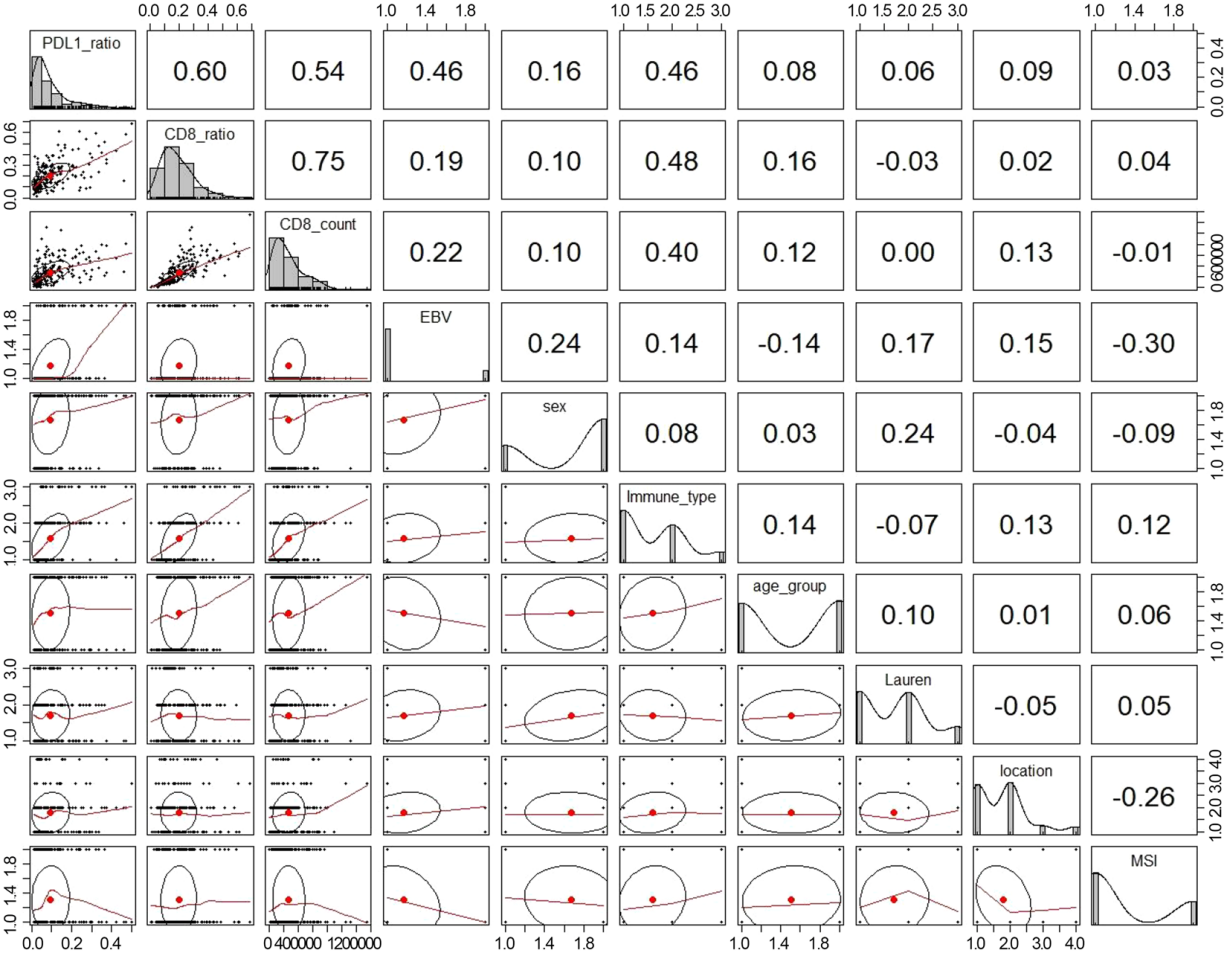
Correlations between PD-L1 ratio and clinicopathologic variables.

Because there was higher correlation between PD-L1 ratio and mRNA level, PD-L1 ratio was used for comparison with clinicopathological variables. PD-L1 ratio correlated most significantly with CD8 ratio (correlation coefficient, r = 0.60), followed by CD8 count (r = 0.54), EBV status, and host inflammatory response (r = 0.46) (Fig. 3).

### Tumor microenvironment subtypes by PD-L1 and CD8 QIA

Because PD-L1 and CD8 ratios by QIA showed high correlation with PD-L1 combined positive score (CPS) and significant survival effects compared to IHC-positive cell counts, we used PD-L1 ratio and CD8 ratio in further analyses. A cut-off value was set with the Maxstat package to divide PD-L1 and CD8 ratio status into two groups: high and low^10^. Of all 241 patients, the high PD-L1 ratio (PD-L1 ratio > 0.136441; PD-L1^H^) group was comprised of 53 cases, and the low PD-L1 ratio (PD-L1^L^, ≤ 0.136441) group was composed of 188 cases. A CD8 ratio of > 0.1636454 was defined as CD8-high (CD8^H^), and 127 patients were identified as CD8^H^. There were 30 (69.8%), 54 (68.4%), and 28 (22.4%) PD-L1^H^ cases of EBV+, MSI-H, and EBV−/MSS GC, respectively.

**Figure 4:**
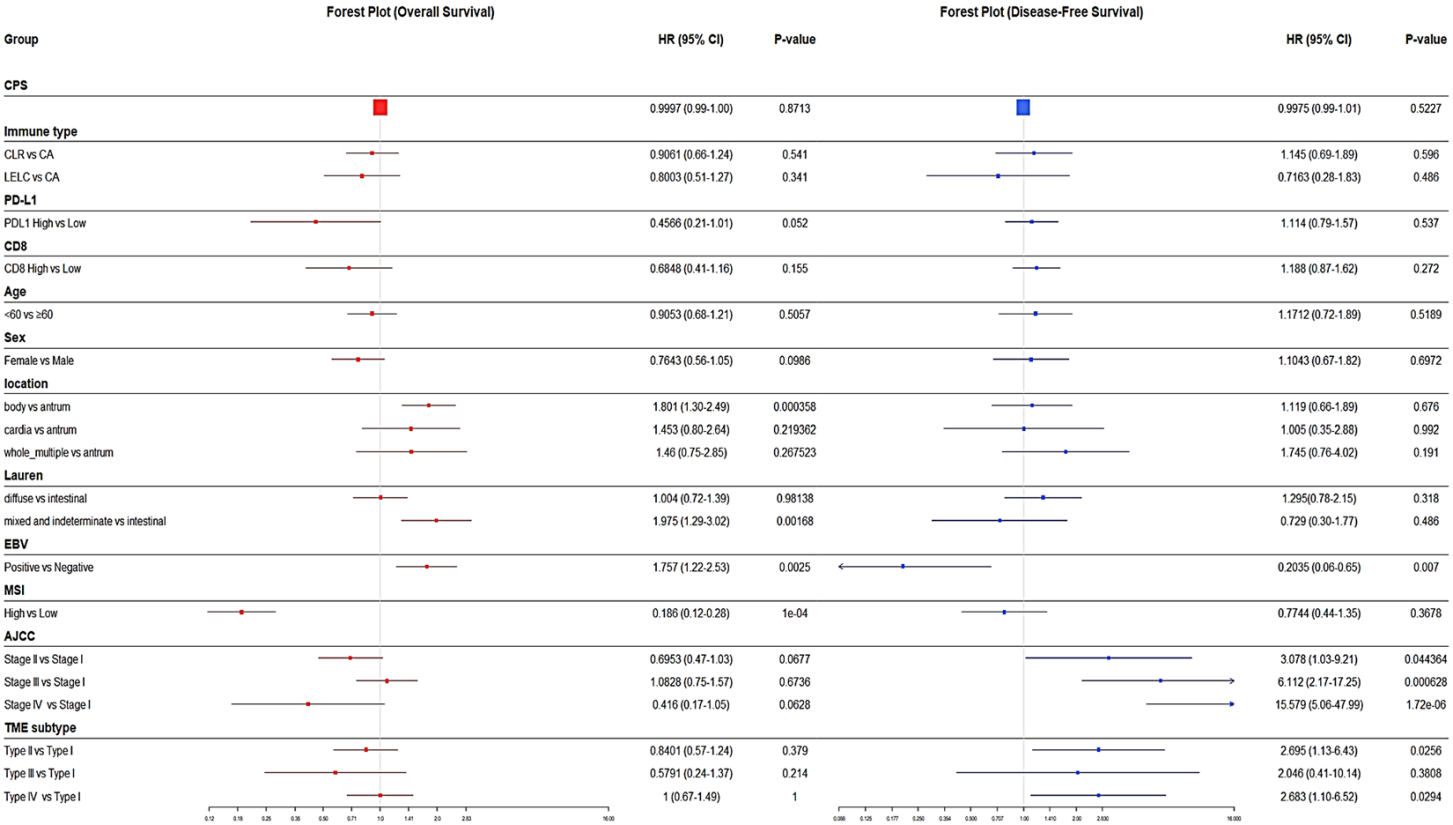
Forest plot of hazard ratio (HR) for disease-free survival and overall survival.

In survival analyses, EBV+ status (P = 0.007) was significantly associated with longer overall survival (OS), while EBV+ (P = 0.0025) and MSI-H (P < 0.001) status were significantly associated with longer disease-free survival (DFS) in univariate analysis (Supplementary Fig. 1). However, these survival differences lost their significance in multivariable Cox regression analyses, except for AJCC stage (P < 0.001). Forest plots of the hazard ratio (HR) for OS and DFS are described in Fig. 4.

**Figure 5:**
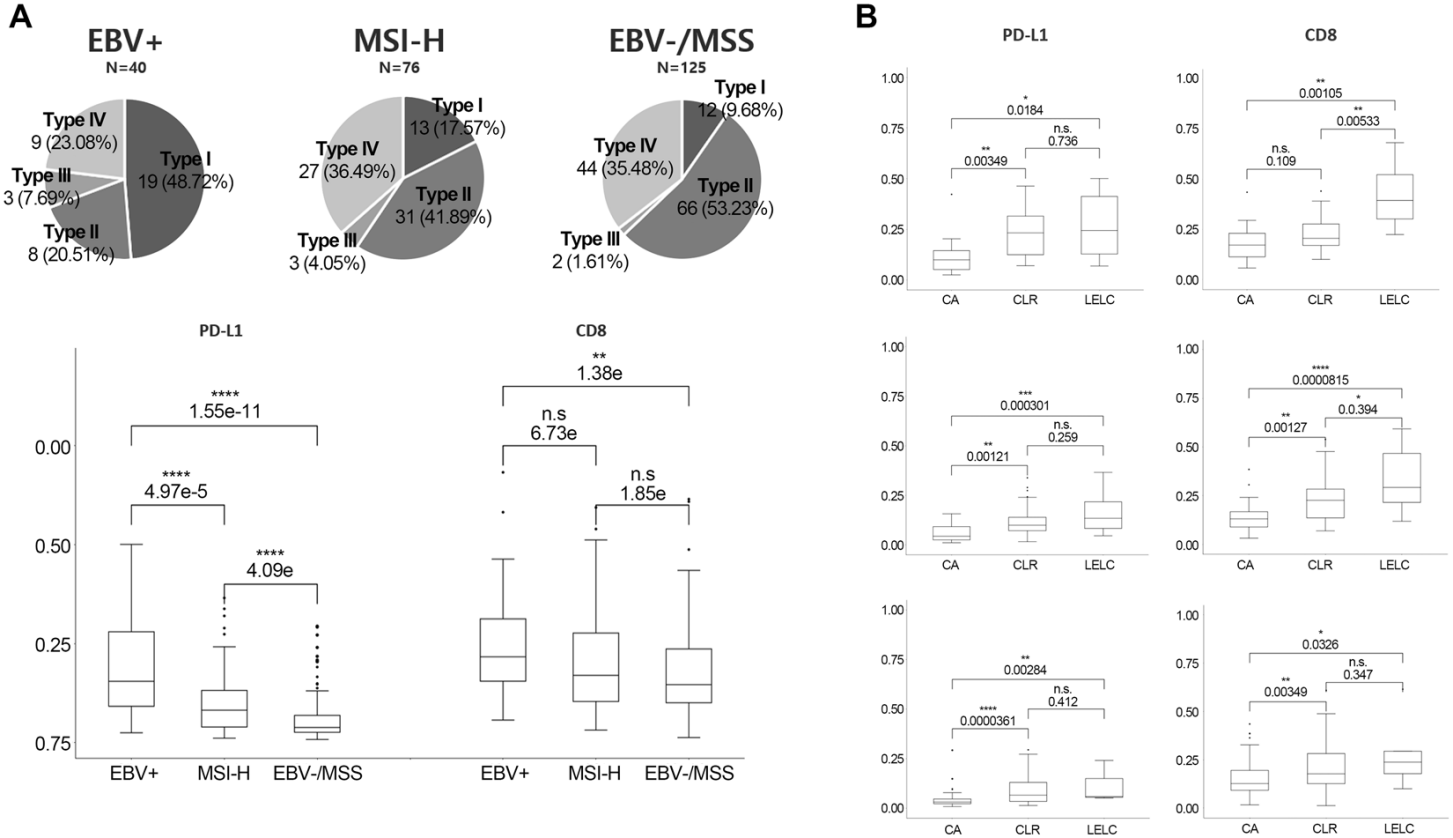
(**A**) Pie charts of TME subtypes based on PD-L1 and CD8 quantitative image analyses (upper) and PD-L1/CD8 ratio in each subtype of gastric cancer (lower). (**B**) PD-L1 and CD8 ratio in relation to host inflammatory responses in EBV+ (upper), MSI-H (middle) and EBV−/MSS gastric cancers.

Based on previously reported melanoma cases^11^ and our previous observations in GC^12^, TME subtypes were classified based on the QIA results of PD-L1-ratio and CD8-ratio. The TME was classified as type I (PD-L1^H^/CD8^H^) in 45 (18.7%) cases, type II (PD-L1^L^/CD8^L^) in 106 (44.0%) cases, type III (PD-L1^H^/CD8^L^) in 8 (3.3%) cases, and type IV (PD-L1^L^/CD8^H^) in 82 (34.0%) cases. The clinicopathological characteristics of the four TME types are summarized in Table 2. Pie charts of TME subtypes based on PD-L1 ratio and CD8 ratio by QIA analyses in each subtype of GC are depicted in Fig. 5A. Figure 5B shows the PD-L1 and CD8 ratios in three distinct subtypes of GC in relation to host inflammatory responses.

**Table 2:** Clinicopathologic parameters in four tumor microenvironment subtypes.

Parameters	Type I (PD-L1^H^/CD8^H^)	Type II (PD-L1^L^/CD8^L^)	Type III (PD-L1^H^/CD8^L^)	Type IV (PD-L1^L^/CD8^H^)	p value
**Tumor microenvironment subtypes**					
	(n = 45)	(n = 106)	(n = 8)	(n = 82)	
age					0.5269
<60	18 (40.0%)	54 (50.9%)	5 (62.5%)	41 (50.0%)	
≥60	27 (60.0%)	52 (49.1%)	3 (37.5%)	41 (50.0%)	
sex					0.2267
male	36 (80.0%)	68 (64.2%)	5 (62.5%)	52 (63.4%)	
female	9 (20.0%)	38 (35.8%)	3 (37.5%)	30 (36.6%)	
location					0.1231
cardia	7 (15.6%)	6 (5.7%)	1 (12.5%)	4 (4.9%)	
body	20 (44.4%)	50 (47.2%)	2 (25.0%)	33 (40.2%)	
antrum	15 (33.3%)	45 (42.5%)	3 (37.5%)	39 (47.6%)	
whole/multiple	3 (6.7%)	5 (4.7%)	2 (25.0%)	6 (7.3%)	
histologic type by Lauren					0.8596
intestinal	18 (40%)	44 (41.5%)	4 (50.0%)	37 (45.1%)	
diffuse	21 (46.7%)	44 (41.5%)	3 (37.5%)	36 (43.9%)	
mixed/indeterminate	6 (13.3%)	18 (17.0%)	1 (12.5%)	9 (11.0%)	
histologic type by host inflammatory response					**3.06E-11**
Conventional	6 (13.3%)	76 (71.7%)	3 (37.5%)	40 (48.8%)	
Crohn-like	25 (55.6%)	28 (26.4%)	5 (62.5%)	32 (39.0%)	
Lymphoepithelioma-like	14 (31.1%)	2 (1.9%)	0 (0.0%)	10 (12.2%)	
EBV					**8.55E-08**
Positive	20 (44.4%)	8 (7.5%)	3 (37.5%)	9 (11.0%)	
Negative	25 (55.6%)	98 (92.5%)	5 (62.5%)	73 (89.0%)	
Microsatellite instability-High					0.8911
Yes	13 (28.9%)	32 (30.2%)	3 (37.5%)	28 (34.1%)	
No	32 (71.1%)	74 (69.8%)	5 (62.5%)	54 (65.9%)	
AJCC/TNM stage					0.3979
I	12 (26.7%)	23 (21.7%)	1 (12.5%)	16 (19.5%)	
II	14 (31.1%)	31 (29.2%)	2 (25.0%)	31 (37.8%)	
III	19 (42.2%)	44 (41.5%)	3 (37.5%)	27 (32.9%)	
IV	0 (0.0%)	8 (7.5%)	2 (25.0%)	8 (9.8%)

TME type I was most significantly associated with LELC and CLR histology (P = 3.0E-11) and EBV+ status (P = 8.55E-08). We also found that TME type IV included 10 cases with LELC histology consisting of two EBV+, five MSI-H, and three EBV−/MSS GC cases. Although EBV+ GC cases were mostly present in type I TME, it is noteworthy that MSI-H GC cases were somewhat scattered throughout diverse TME subtypes; 17.1% were type I, 42.1% were type II, 4% were type III, and 36.8% were type IV. In survival analyses, patients with type I TME showed a longer OS compared to patients with type IV TME (P = 0.0294).

## Discussion

In this study, we examined archived tumor specimens obtained from a large cohort of patients with stage IB-IV GC for IHC expression of PD-L1 and CD8 in three distinct subtypes of GC (EBV+/MSI-H/EBV− and MSS) by QIA, and correlated them with mRNA expression level and manual interpretation. Both PD-L1 ratio and CD8 ratio correlated well with their respective mRNA levels and manual interpretation results. The PD-L1 ratio correlated well with CD8+ immune cells and TME subtype by QIA, and also correlated well with host inflammatory response and EBV+ GC.

Therapies blocking the PD1/PD-L1 axis have resulted in different rates of tumor response for a variety of cancer types^13^. In GC, ~50% of EBV+ and ~60% of MSI subgroups show high levels of PD-L1 expression, highlighting the molecularly defined patient population most likely to derive benefit from immune checkpoint blockade^14–16^. Recent meta-analyses have shown GCs with high PD-L1 expression had deeper tumor infiltration (pT) and were more positive for lymph node metastasis (pN), suggesting that PD-L1 is expressed through oncogenic stimulation and is associated with poorer prognosis^11,12,17^. Given that high PD-L1 expression is closely associated with EBV+ and MSI-H GC, PD-L1 may also be expressed through adaptive immune resistance, which occurs due to the many cytotoxic T-cells and neoantigens present within tumor cells (secondary to EBV itself and the many frameshift mutations seen in EBV+ and MSI-H cancers) and is associated with favorable prognosis. These contradictory results suggest that PD-L1 expression in GC is caused either by oncogenic stimulation resulting in poor prognosis and by adaptive immune resistance affecting favorable prognosis bringing favorable prognosis, and those results are consistent with previous observations in melanoma^18^.

Classifying GC based on PD-L1 expression and TIL may be more complex than the initial morphological studies performed in melanoma using IHC analyses suggest, and will likely require quantitative and special determination to be used as highly predictive tools to define optimal therapy for patients with advanced cancers^11^. In the present study, we performed PD-L1 IHC with SP142, an FDA-approved antibody, in whole sections of 241 surgically resected GCs. The cases included many EBV+ (N = 40) and MSI-H (N = 76) GCs, and the study measured PD-L1 with QIA to assess its clinical significance and possible utility in pathology. In our previous study on TME classified by manual interpretation of PD-L1 and CD8 using the same IHC-stained slides, type I (PD-L1^H^ and CD8^H^) showed the best survival and was enriched with EBV+ and MSI-H GC cases. The classification of TME groups in The Cancer Genome Atlas (TCGA) data based on PD-L1 and TIL status, the type I TME was associated with oncogenic viral infection^19^. In the present study, we found similar results to these TCGA results and our previous analyses: EBV+ GC cases are mostly type I TME, while MSI-H GC cases are somewhat scattered, with a diversity of TME subtypes^19^. Various expression patterns of PD-L1 and their prognostic significance have also been reported^20,21^. We recently found that all EBV+ GCs with PD-L1 expression responded dramatically to pembrolizumab; however, not all MSI-H GCs express PD-L1, and only patients with PD-L1-expressing GC responded well to pembrolizumab. PD-L1 expression was the most useful of the predictive markers used in that study^22^.

In this study, we introduced QIA to interpret the IHC results of PD-L1 and CD8, and the results were directly compared with manual interpretation results and mRNA expression levels. Given that manual interpretation has limitations, such as low reproducibility and high inter-observer variation, analysis by QIA can reduce inter-observer variation, provide more objective and unbiased assessments, and improve the reproducibility of interpretation. Moreover, it can facilitate the collection of large amounts of data for statistical analysis and increase the efficiency of pathological interpretation by assessing numerous samples in a short time. In the present study, QIA of IHC-stained, whole-slide images correlated very well with microscopic interpretation of PD-L1 and mRNA levels. Moreover, the CD8 ratio significantly correlated with CD8 mRNA levels. Our results highlight the positive aspects of QIA for digital images. Future work should focus on deep assessment of IHC-stained images with integrated regional information.

Although this study was the first to analyze PD-L1 and CD8 expression status by QIA in a large cohort of GC cases, it has some limitations. First, selection bias is possible because this is a retrospective study, and no patients were treated with immunotherapy. Second, although SP142 is an FDA-approved antibody, this clone was developed for atezolizumab biomarker assays for urothelial carcinoma and non-small cell lung cancers^8^. To overcome these limitations, we are planning future stratification studies on GC patients with known responses to immunotherapy.

In conclusion, QIA correlated well with mRNA levels and the manual interpretation results of IHC. We successfully classified four TME groups of GC based on the QIA results of whole digitalized slide images for PD-L1 and CD8 expression status. These results demonstrate that QIA can be used as a diagnostic tool to classify TME subtype.

## Material and Methods

### Patient selection

Patients who underwent surgery for primary gastric carcinoma from September 2004 to May 2012 at the Samsung Medical Center were eligible for this study. Among them, 40 EBV− positive cases, 76 MSI-H cases, and 125 EBV(−)/MSS cases were selected, for a total of 241 patients with GC, as previously described^12^. No patient had received preoperative chemotherapy and/or radiation therapy or had any other uncontrolled cancer at the time of GC diagnosis. The mean follow-up period was 48.4 ± 20.1 months. Four patients developed other organ cancers (lung, skin, pancreas, and colon) during the follow-up period. Clinical data, including demographic features, pathological characteristics, and treatment outcomes, were obtained by reviewing medical records, and information on deaths was provided by the national statistical office. Tumor stage was defined according to TNM classification as described in the 8th edition of the AJCC cancer staging manual^23^. Informed consent was obtained from all patients and the Institutional Review Board of the Samsung Medical Center approved this study. This study was carried out in accordance with the approved guidelines.

### Immunohistochemistry and quantitative image analysis

IHC was performed on each representative whole section from all 241 formalin-fixed paraffin-embedded (FFPE) GC samples. Staining for PD-L1 was conducted using a Food and Drug Administration (FDA)-approved rabbit anti-human PD-L1 monoclonal antibody (clone SP142; Ventana, Tucson, AZ, USA). The percentages of tumor cells and peritumoral immune cells that stained positive for PD-L1 (combined positive score: CPS) were analyzed independently by two pathologists (J.C. and K.M.K.). Staining for CD8 in FFPE tissue sections was conducted using a CONFIRM-anti-CD8 (SP57) rabbit monoclonal primary antibody without dilution with Ventana BenchMark XT via the OptiView DAB IHC Detection Kit (Catalog Number 760–700; Ventana).

All IHC-stained slides were digitally scanned at 20x magnification using a ScanScope Aperio AT Turbo slide scanner (Leica Microsystems). The computational whole slide digital image workflow encompasses three steps: (1) image preprocessing, (2) nuclei segmentation, and (3) quantitative image analysis. In image preprocessing, a color deconvolution technique^24^ was used to separate the pure DAB and hematoxylin-stained areas, leaving a complimentary image. Hematoxylin-stained images were then used for cell segmentation based on our segmentation algorithm^25^. The chromogenic intensity in DAB-stained images was then quantified (i.e., for PD-L1 and CD8). In the quantitative image analysis, single cell-based information, including pixel intensity and cell size measurements (i.e., pixel area), was visualized using a bivariate scatter plot (Fig. 1 shows an example). In addition, the chromogenic signal intensity was quantitatively measured, which is analogous to the fluorescence-activated cell sorting data obtained in flow cytometry^26^. Thresholds for cell type identification (i.e., positive/negative) were determined based on the distribution of scatter plots, and the same value was used across all samples. The PD-L1/CD8 positive cell ratio was defined as the absolute number of positive cells per total number of cells.

### PD-L1 and CD8 mRNA analysis

Total RNA extracted from the available GC FFPE specimens was used to measure PD-L1 and CD8 gene expression with nCounter® Gene Expression Human Immunology Panel containing 494 human immune signature genes (NanoString Technologies, Seattle, WA, USA). For all selected cases, archival tissue was available for RNA extraction, with an estimated tumor cell percentage over 60% after dissection under microscopy. Total RNA (200 ng) was extracted from three to four 4-μm-thick tissue sections of representative primary tumor blocks using a High Pure RNA Paraffin kit (Roche Diagnostic, Mannheim, Germany). RNAs were hybridized to target sequence-specific capture probes and fluorescent-labeled reporter probes. The mRNA-probe complexes were washed, immobilized, and quantified by fluorescence imaging as previously described^27,28^, and part of the results were published previously^29^.

### Statistical analysis

The clinicopathological characteristics of patients, such as age, sex, pTNM stage (AJCC 7th edition), disease-free survival (DFS), and overall survival (OS), were analyzed. The SPSS 18.0 statistical software program (SPSS Inc., Chicago, IL, USA) was used for statistical analyses. PD-L1 expression status, CD8 expression status, and clinicopathological variables were compared using Pearson’s chi-squared test, and the results were further compared using a linear-by-linear association. The mean values of PD-L1 ratio and CD8 ratio were compared using a Kruskal-Wallis test. The Kaplan-Meier method was used to estimate DFS and OS. To evaluate the associations between clinicopathological factors and survival, a Cox proportional hazard model was used. P-values less than 0.05 were considered statistically significant.

## Electronic supplementary material

Figure S1
