# Supplementary material for: Computational measurement of tumor immune microenvironment in gastric adenocarcinomas
Source: Sci Rep. 2018 Sep 17;8:13887. doi: 10.1038/s41598-018-32299-0 (PMC6141531; doi:10.1038/s41598-018-32299-0)

Supplementary Figures and Information

Computational measurement of tumor immune microenvironment in gastric adenocarcinomas

Young Hwan Chang[1*], You Jeong Heo[2*], Junhun Cho[3*], Sang Yong Song[3], Jeeyun Lee[4] and Kyoung-Mee Kim[3]

[1]Department of Biomedical Engineering and Computational Biology Program, Oregon Health and Science University (OHSU), Portland, OR 97239 USA

[2]The Samsung Advanced Institute for Health Sciences & Technology (SAIHST), Samsung Medical Center, Sungkyunkwan University School of Medicine, Seoul, Korea

[3]Department of Pathology & Translational Genomics, Samsung Medical Center, Sungkyunkwan University School of Medicine, Seoul, Korea

[4]Division of Hematology-Oncology, Department of Medicine, Samsung Medical Center, Sungkyunkwan University School of Medicine, Seoul, Korea

Figure S1. Kaplan-Meier survival analyses of PD-L1 ratio, EBV+, MSI-H, and tumor microenvironment subtypes: A) Overall survival analysis and B) Disease-free survival analysis.

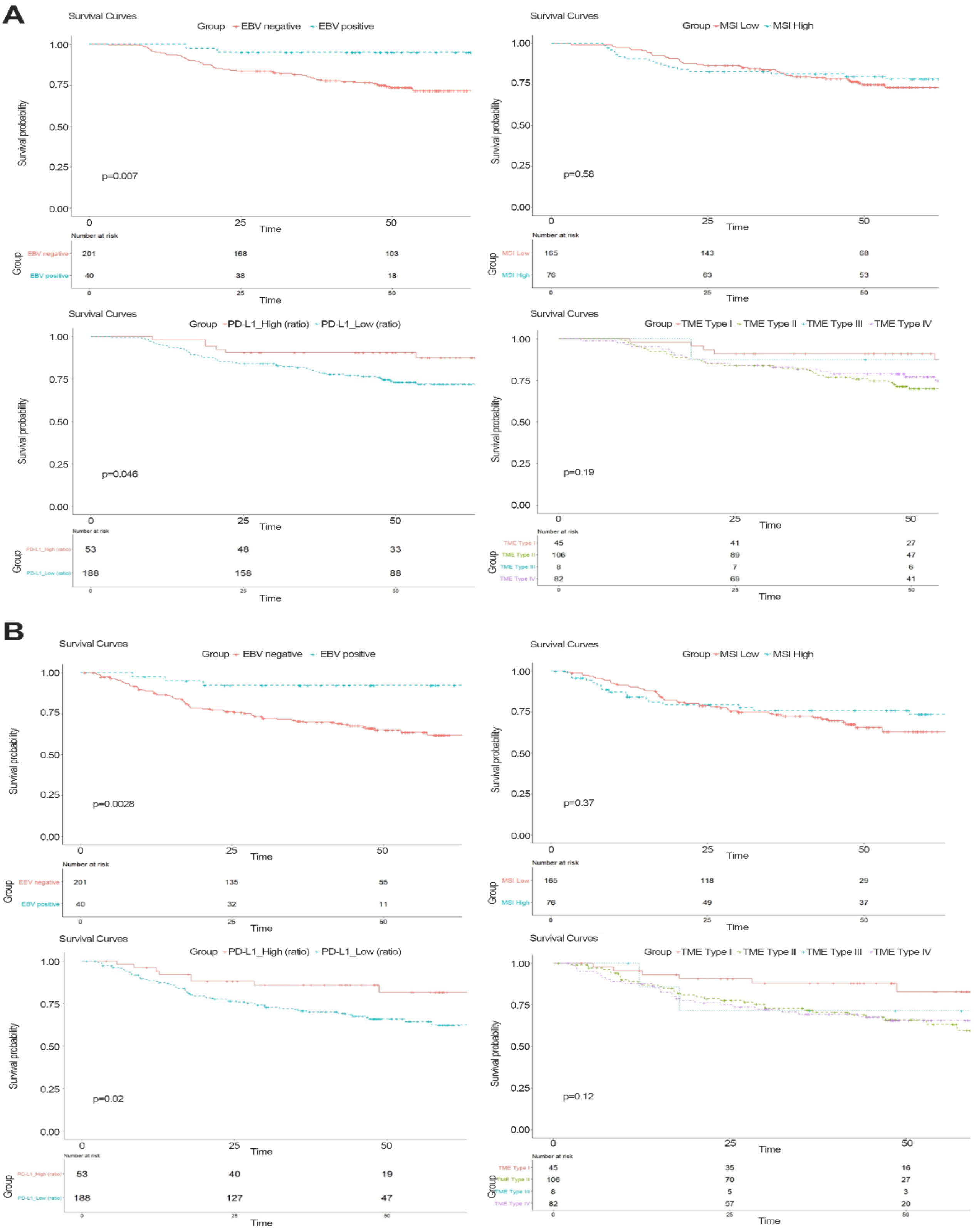

Supplement: Supplementary file 1 — Figure S1 [file 41598_2018_32299_MOESM1_ESM.pdf]
